# A Mild Aqueous Sonogashira Reaction as a Fluorescent Labeling Strategy for 5-Bromide-2′-Deoxyuridine

**DOI:** 10.3390/molecules23010154

**Published:** 2018-01-12

**Authors:** Shufang Wang, Yongxin Gao, Shigang Shen, Hui Wen, Huaqing Cui

**Affiliations:** 1 State Key Laboratory of Bioactive Substances and Function of Natural Medicine, Institute of Materia Medica, Peking Union Medical College and Chinese Academy of Medical Sciences, Beijing 100050, China; wangshufang@imm.ac.cn (S.W.); wenhui@imm.ac.cn (H.W.); 2 Beijing Key Laboratory of Active Substances Discovery and Drugability Evaluation, Institute of Materia Medica, Peking Union Medical College and Chinese Academy of Medical Sciences, Beijing 100050, China; gaoyongxin@imm.ac.cn; 3 Key Lab of Analytical Science and Technology of Hebei Province, College of Chemistry and Environmental Science, Hebei University, Baoding 071000, China; shensg@hbu.edu.cn

**Keywords:** Sonogashira reaction, BrdU, aqueous reaction, fluorescent labeling

## Abstract

C5-modified uridines are a valuable class of nucleoside analogues, both as potent chemotherapy agents and through their use as the conjunction site in DNA labeling strategies. As an important C5-modified uridine, BrdU has been used in cell proliferation assays since the 1980s. Currently, the detection of BrdU relies on traditional immunostaining; however, this approach has its limitations. Thus, it is desirable, albeit difficult, to develop chemistry methods to fluorescently label BrdU in a cellular context. In the present study, we report our efforts toward developing a robust chemistry methodology for BrdU fluorescent labeling. The Sonogashira reaction was chosen as the key reaction, and various alkynyl groups (aliphatic or aryl) containing fluorescent dyes were synthesized to cross-couple with BrdU. Various bases and catalyst systems were screened to evaluate the optimum conditions. A mild aqueous Sonogashira reaction (K_2_PdCl_4_, S-Phos, *n*-Bu_4_N^+^OH^−^, Sodium d-isoascorbate, EtOH/H_2_O = 1:1, 37 °C, Ar) was obtained to enable high-yielding BrdU fluorescent labeling.

## 1. Introduction

The C5-modified uridines are a group of valuable nucleoside analogues utilized in biomedical research [1,2]. Several C5-modified uridines, such as 5-bromo-2′-deoxyuridine (BrdU) [3], 5-ethynyl-2′-deoxyuridine (EdU) [4,5], and 5-vinyl-2′-deoxyuridine (VdU) [6] are commonly used in cell proliferation assays, in which they initially incorporate into cellular DNA and then serve as the important conjunction site for DNA labeling. In addition, there are various C5-modified uridines which have been proven to have potent anticancer or antiviral activities and serve as important chemotherapeutic agents [2]. Considering the biological importance of C5-modified uridines, the chemistry community is continuously developing novel methods to efficiently label C5-modified uridines under mild, biologically relevant conditions [7,8].

BrdU was the first C5-modified uridine to be routinely used in cell proliferation assays [3]. BrdU can efficiently incorporate into DNA during the S-phase of the cell cycle, and was widely used to investigate DNA replication in many biological experiments [9]. The conventional method to detect cellular BrdU relies upon antibody staining; however, this requires DNA denaturation [9]. The need for these harsh DNA denaturation conditions typically results in numerous problems including destroying the protein antigenic determinant, as well as limiting the concurrent detection of proteins. Moreover, the specificity of commercial BrdU antibodies is often reported to be problematic, and the BrdU antibody can falsely recognize other C5-modified uridines, such as EdU [10,11].

EdU and VdU were subsequently developed to solve these limitations and to replace BrdU in cell proliferation assays [8]. In particular, both EdU and VdU are recognized via bioorthogonal reactions, thus removing the need for antibody immunostaining with unspecific antibodies. Over the past decade, there has been a tendency to incorporate two or three C5-modified uridines (BrdU, EdU, and VdU) into the same chromosome or tissue in order to accurately trace cell development [12,13]. To facilitate this approach, it is essential to develop a chemical method to label cellular BrdU, instead of relying upon the conventional antibody method. In particular, antibody staining is often performed under physiological conditions, while bioorthogonal reactions often require the use of carefully chosen bases, acids, and other catalysts, which may interrupt the binding affinity of an antibody. Unfortunately, a robust chemical method for fluorescently labeling cellular BrdU has proven difficult to develop [14]. To date, limited results have been reported in the literature; however, recently, researchers have explored a mild and specific Suzuki–Miyaura reaction to label 5-iodo-2′-deoxyuridine (IdU), taking advantage of the fact that IdU is more chemically reactive than BrdU [14].

In this study, we wanted to develop a mild and efficient set of reaction conditions to allow us to label BrdU with a fluorescent tag. The Sonogashira cross-coupling reaction is the palladium-catalyzed coupling of terminal alkynes with aryl halides, and is an attractive and powerful tool for the formation of C–C bonds [15,16,17,18,19]. The Sonogashira reaction is generally performed in the presence of a palladium source and uses CuI as a co-catalyst in various organic solvents [16,18,20,21]. Importantly, it has been shown that the Sonogashira reaction can be carried out in aqueous media [17], which opens up the possibility of using it in a biological setting. Therefore, we decided to investigate the application of the Sonogashira reaction to labelling BrdU with fluorescent tags. Once the BrdU Sonogashira labeling method has been successfully optimized, this condition can be possibly adapted to the fluorescent labeling of cellular incorporated BrdU in fixed cells.

## 2. Results and Discussion

### 2.1. Design and Synthesis of Various Aliphatic or Aryl Alkyne-Containing Fluorescent Dyes

**Figure 1:**
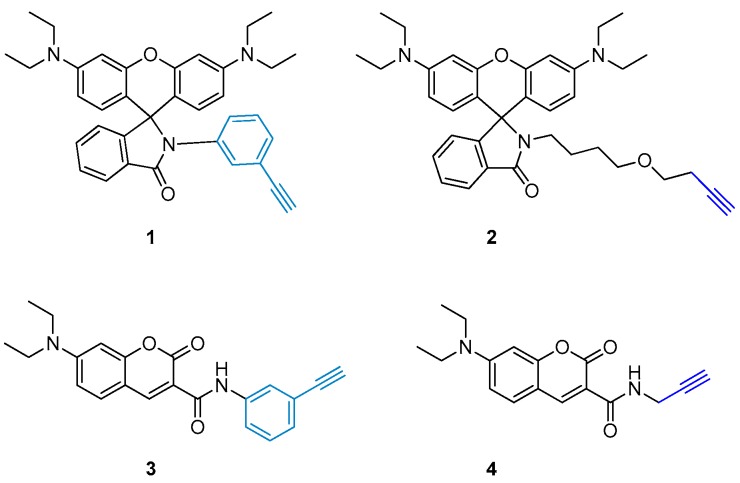
The chemical structures of four fluorescent probes used in this study. **1** and **2** are rhodamine B-based fluorescent probes. **1** contains a terminal aliphatic alkynyl group, and **2** has a terminal aryl alkynyl group. **3** and **4** are coumarin-based fluorescent probes. **3** contains a terminal aliphatic alkynyl group, and **4** has a terminal aryl alkynyl group.

The Sonogashira reaction requires one of the coupling partners to have a terminal alkyne group. Therefore, before we could investigate the reaction conditions for the cross-coupling, we required access to the alkynyl-containing fluorescent probes which would be used to label BrdU. For this study, we designed and synthesized four alkyne-containing fluorescent probes. We choose two commonly used fluorophore cores—rhodamine B and coumarin—which have excellent fluorescence quantum yields but, importantly, exhibit different physical and chemical properties such as water solubility [22,23,24,25]. Both of the probes were functionalized with terminal alkyne groups to be used as the reactive handle in the Sonogashira reaction. Secondly, it is possible that aliphatic or aryl alkynyl groups exhibit differing reactivity in the Sonogashira coupling reaction with BrdU; therefore, we chose to include both moieties in the study (Figure 1).

**Scheme 1:**
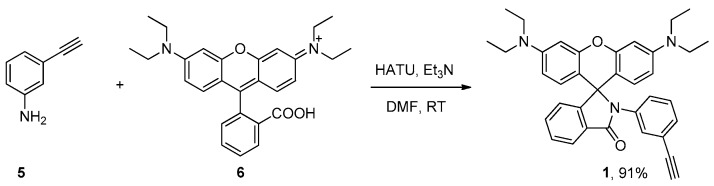
The synthesis of fluorescent probe **1**: HATU (2-(7-Azabenzotriazol-1-yl)-*N*,*N*,*N*′,*N*′-tetramethyluronium hexafluorophosphate), Et_3_N, *N*,*N*-Dimethylformamide, 16 h, Room Temperature.

Next, we carried out the synthesis of the four designed fluorescent probes. The synthesis of probe **1** was straightforward, as shown in Scheme 1. 3-Ethynylaniline reacted with rhodamine B to form the lactam in the presence of HATU and triethylamine. This reaction was completed after 16 h at room temperature and afforded **1** with excellent yield.

**Scheme 2:**
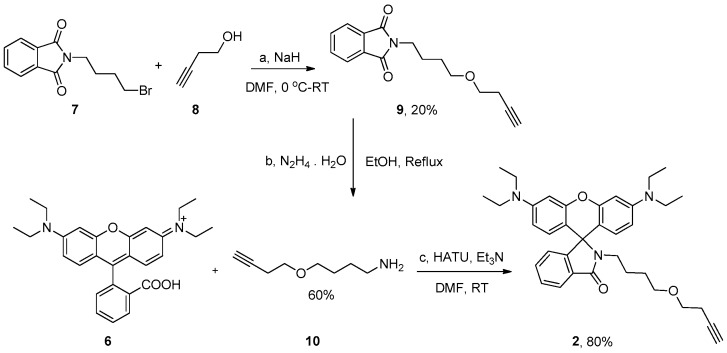
The synthesis route of fluorescent probe **2**. a: 1.2 eq. NaH, DMF, 2.5 h, 0 °C to RT; b: 10 eq. N_2_H_4_·H_2_O, EtOH, reflux, 0.5 h; c: HATU, Et_3_N, DMF, 12 h.

The synthesis of the aliphatic alkyne-containing rhodamine B probe was carried out as shown in Scheme 2. We reasoned that a long aliphatic chain incorporated into the structure of the probe could reduce the potential steric hindrance stemming from the rhodamine B core. The alkyl chain portion was prepared from *N*-(4-bromobutyl) phthalimide **7** and 3-butyn-1-ol **8** in the presence of NaH in DMF. The subsequent adduct **9** was deprotected with hydrazine to afford the requisite amine in good yields. We noted during the course of this reaction that reduction of the triple bond could also occur and, therefore, careful monitoring of the reaction is necessary for reproducible yield. Finally, we coupled the primary amine **10** and rhodamine B **6** to afford probe **2** in good yield and high purity.

**Scheme 3:**
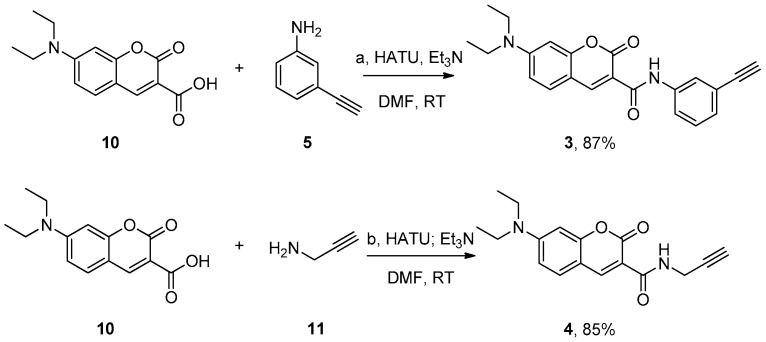
The synthesis of fluorescent probe **3** and **4**: a, HATU, Et_3_N, DMF, 14 h; b, HATU, Et_3_N, DMF, 14 h.

The syntheses of the coumarin-based fluorescent probes **3** and **4** are described in Scheme 3. 2-Ethynylaniline **5** reacted with fluorescent dye **10** to produce fluorescent probe **3** in excellent yield. The synthesis of probe **4** was completed from 2-propynylamine **11** and coumarin **10**.

**Figure 2:**
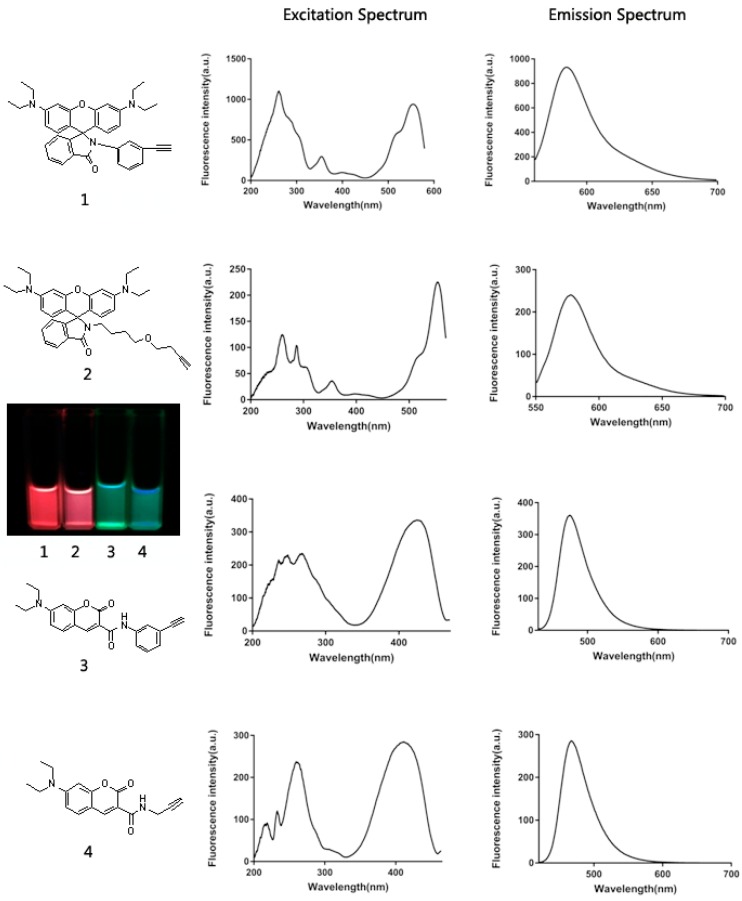
The fluorescence spectra of the four fluorescent probes used in this study. All of the four probes were dissolved in methanol at the concentration of 10 μM. **1** and **2** are rhodamine B-based fluorescent probes, which have red color fluorescence. **3** and **4** are coumarin-based fluorescent probes, which have green color fluorescence.

The four fluorescent probes were checked for fluorescent properties. To obtain the fluorescence excitation and emission spectra, compounds **1**, **2**, **3** and **4** were dissolved in methanol at the concentration of 10 μM. The fluorescence spectrums were scanned with a fluorospectrophotometer, and the maximum excitation and emission wavelengths were recorded in Figure 2. The methanol solutions of the four probes were also imaged under UV light to show the color of the fluorescence (Figure 2).

In summary, we synthesized four alkyne-containing fluorescent probes (**1**–**4**, Figure 1), which will be used as one of the coupling partners in the Sonogashira reaction. The probes include two different fluorophores—rhodamine B and coumarin—as this provides probes with varied physical and chemical properties such as water solubility and differences in steric hindrance. In addition, the terminal aliphatic or aryl alkynyl group might also affect the reactivity towards the Sonogashira reaction.

### 2.2. Optimization of Sonogashira Reaction Conditions

**Scheme 4:**
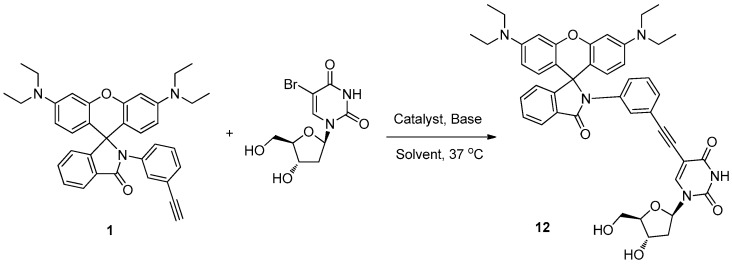
The Sonogashira reaction between **1** and BrdU. The metal source, the base, and the solvent are three factors that need to be optimized in order to find the optimum conditions.

We decided to use probe **1** as the model substrate to develop and optimize the conditions necessary to mediate the Sonogashira coupling condition between the probe and BrdU (Scheme 4). In order to ensure that the protocol is applicable for use in biological settings, we focused our attention on developing mild reaction conditions in order to conserve important cellular residues. In order to become widely used, and considering the fact that the majority of BrdU fluorescent labeling often occurs in an aqueous medium, we chose water as the solvent of choice in the reaction. However, it has been noted by others that it is important that the coupling partners are fully dissolved for the reaction to be successful; therefore, we focused our attention on binary mixtures of organic solvents and water. In addition to the solvent, two factors require optimization for the reaction to be successful: the source of the palladium metal and the base used. 

Initially we investigated the effect of the base on the success of the Sonogashira reaction (BrdU and Probe **1**). The reaction was carried out under general Sonogashira conditions [16,20] in the presence of various bases. We utilized a 1:1 mixture of DMF and water as solvent and K_2_PdCl_4_/DTBPPS (3-(Di-*tert*-butylphosphonium)propane sulfonate) as the catalytic system (1:1, 20% equivalent of BrdU). The general procedure is as follows. Under an argon atmosphere, BrdU, the base (3 equivalent of BrdU), and sodium d-isoascorbate (1.5 equivalent of BrdU, which was used to keep the system anaerobic) [26] were added to the vessel followed by the addition of water; the mixture was then stirred for 5 min. K_2_PdCl_4_ and DTBPPS were then added and the mixture was stirred for 2 h at 37 °C. The required alkyne (1 equivalent of BrdU) and CuI (10% equivalent of BrdU) in DMF were then added to the reaction mixture and stirred at 37 °C until LCMS (Liquid chromatograph-mass spectrometer) analysis showed complete conversion to the product. We chose several bases from previously reported Sonogashira reactions, including Na_2_CO_3_, Cs_2_CO_3_, Quinine, DABCO (1,4-Diazabicyclo[2.2.2]octane), Et_3_N and *n*-Bu_4_N^+^OH^−^. The results below in Table 1 show that using quinine, DABCO, and *n*-Bu_4_N^+^OH^−^ as the base afforded the desired product in higher yields. Among these bases, *n*-Bu_4_N^+^OH^−^ exhibits the best water solubility, and, therefore, we decided to use *n*-Bu_4_N^+^OH^−^ as the base going forward.

With the base in hand, we next turned our attention to the choice of catalyst and ligand for the reaction. Several catalyst systems have been reported for Sonogashira reactions in aqueous media. When screening for the catalyst, BrdU, probe **1**, and *n*-Bu_4_N^+^OH^−^ (1:1:3) were used as the model conditions. For the Palladium source, Pd(OAc)_2_ and K_2_PdCl_4_ are the classical options that have been commonly used in various Sonogashira coupling reactions [17,20]. We initially tested Pd(OAc)_2_, DTBPPS, and CuI as the catalyst in a mixture of DMF/H_2_O (1:1) which produced the product in 22% yield (Table 2). Upon changing the source of palladium to K_2_PdCl_4_, we noted two-fold increase in the isolated yield. This is partially due to the better aqueous dissolution of K_2_PdCl_4_ when compared to that of Pd(OAc)_2_. We then turned our attention to the ligand used in the system. We were aware that S-Phos has found great success in mediating the Sonogashira reaction and it comes with the added benefit of increased solvent solubility and stability over DTBPPS. Traditionally, the metals of Palladium and copper are both needed in a Sonogashira reaction as the catalysts [20]. CuI in the catalyst system can facilitate the in situ formation of Copper acetylide. However, the presence of CuI can also induce undesired side products. The optimization of the ligand system and the base can help to stabilize the ionic intermediates of the catalytic cycle, and, under these circumstances, the Sonogashira reaction can proceed well without the presence of CuI [17,20]. It is important to note here that the use of S-Phos precludes the need for the CuI co-catalyst. We switched the solvent used in the reaction, due to the increased solubility of the ligand, to a 1:1 mixture of EtOH/H_2_O, and were delighted to isolate the requisite cross-coupled product in 75% after 4 h (Table 2).

In summary, we have optimized the key Sonogashira reaction between BrdU and a terminal alkyne (K_2_PdCl_4_, S-Phos, *n*-Bu_4_N^+^OH^−^, Sodium d-isoascorbate, EtOH/H_2_O = 1:1, 37 °C, argon protection). The reaction is fast and can reproducibly afford the product in high yields in less than 4 h. Moreover, contrary to the more traditional conditions, CuI is not required in this Sonogashira reaction. 

### 2.3. Labeling BrdU with Alkyne-Containing Fluorescent Dyes

**Figure 3:**
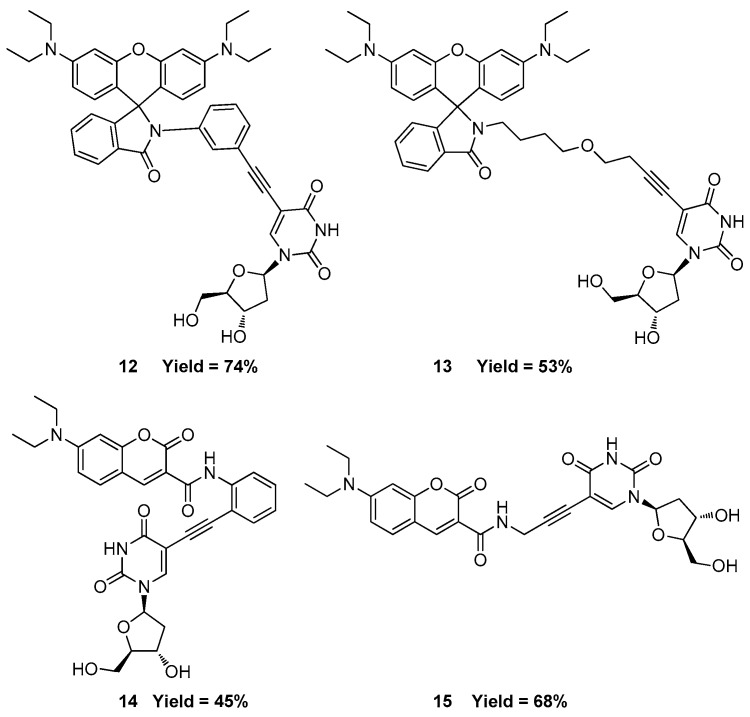
The BrdU fluorescent labeling rates of the four alkynyl groups under our Sonogashira reaction conditions.

With these optimized conditions in hand, we were ready to label BrdU with various fluorescent probes. We were particularly interested in evaluating the different reactivity profiles of the aliphatic alkynyl group versus the aryl alkynyl group. Under the now-optimized conditions, all four fluorescent probes can achieve reasonable BrdU labeling (Figure 3). However, there is no obvious advantage between the aliphatic or aryl alkynyl groups regarding the reactivity. In addition, in order to mimic the complex cellular labeling environment, we also performed the BrdU Sonogashira labeling with the presence of four deoxynucleosides (A, T, C, G). The substrate cocktail contained the same concentrations of all four deoxynucleosides and BrdU. Indeed, in cultured cells, a halogen-modified nucleoside is almost undetectable unless they are intentionally added to the culture medium. In our substrate cocktails, BrdU is the only halogen-modified nucleoside. We found that the existence of the other four deoxynucleosides did not affect the process of the BrdU Sonogashira labeling.

## 3. Materials and Methods

### 3.1. General Information

All reagents and anhydrous solvents are commercially available and were used without further purification. Temperatures are given in degrees Celsius (°C). NMR spectra were recorded on JEOL ECZ-400S spectrometers (JEOL, Ltd., Tokyo, Japan) using TMS (Tetramethylsilane) as the internal standard. The chemical shifts are reported in δ units (ppm) and the coupling constants (*J*) are in Hertz (Hz). The following multiplicity abbreviations are used: (s) singlet, (d) doublet, (t) triplet, (q) quartet, (m) multiplet, and (br) broad. ESIMS (Electrospray ionization-tandem mass spectrometry) data were measured on an Agilent 1100 Series (Agilent Technologies, Palo Alto, CA, USA). All reactions were monitored by TLC. Column chromatography was carried out using silica gel (Qingdao Marine chemical, Qingdao, China) with a 200~300 mesh size. Flash column chromatography was performed on a Biotage Isolera One (Uppsala, Sweden). Purity was determined by LCMS (Agilent Technologies, Palo Alto, CA, USA) and NMR spectroscopy (JEOL ECZ-400S, Tokyo, Japan). All of the final compounds were of purity higher than 95%.

### 3.2. Synthesis of 3′,6′-Bis(diethylamino)-2-(3-ethynylphenyl)spiro[isoindoline-1,9′-xanthen]-3-one (***1***)

3-Ethynyl-benzenamin (351.4 mg, 3 mmol) was added to a suspension of Rhodamine B (956.0 mg, 2 mmol), HATU (1.141 g, 3 mmol), and Et_3_N (809.5 mg, 8 mmol) in DMF (15 mL) at room temperature for 16 h. Then, ethyl acetate and water were added. The separated aqueous phase was extracted with ethyl acetate (3 × 30 mL). The combined organic phases were washed with water, dried with Na_2_SO_4_, and evaporated to dryness. Purification was carried out with silica gel (hexane/ethyl acetate = 4:1) to get the white solid **1**. ESI/MS: 542.29 [M + H]^+^. ^1^H-NMR (400 MHz, DMSO-*d*_6_) δ 7.85 (d, *J* = 7.9 Hz, 1H), 7.53 (d, *J* = 1.9 Hz, 2H), 7.15 (s, 2H), 7.04 (d, *J* = 8.6 Hz, 1H), 6.92 (s, 1H), 6.82–6.74 (m, 1H), 6.51 (d, *J* = 8.7 Hz, 2H), 6.35 (d, *J* = 11.5 Hz, 2H), 6.26 (s, 2H), 4.08 (s, 1H), 3.26 (m, 8H), 1.05–1.00 (m, 12H).

### 3.3. Synthesis of 2-(4-(But-3-yn-1-yloxy)butyl)isoindoline-1,3-dione (***9***)

*N*-(4-bromobutyl)phthalimide(1.410 g, 5 mmol) and 3-butyn-1-ol (525.8 mg, 7.5 mmol) were dissolved in dry DMF (15 mL). NaH (144.0 mg, 6 mmol) was added in small portions at 0 °C. After 3 h, the reaction was quenched by NH_4_Cl solution and extracted with ethyl acetate (3 × 50 mL). The combined organic phases were washed with water, dried with Na_2_SO_4_, and evaporated to dryness. Purification was carried out with silica gel (hexane/ethyl acetate = 3:1) to get the white solid intermediate **9**. ESI/MS: 272.12 [M + H]^+^. ^1^H-NMR (400 MHz, DMSO-*d*_6_) δ 7.91–7.79 (m, 4H), 3.58 (s, 2H), 3.43 (s, 2H), 3.40 (s, 2H), 2.76 (s, 1H), 2.39–2.33 (m, 2H), 1.62 (d, *J* = 7.7 Hz, 2H), 1.51 (d, *J* = 8.7 Hz, 2H).

### 3.4. Synthesis of 2-(4-(But-3-yn-1-yloxy)butyl)-3′,6′-bis(diethylamino)spiro[isoindoline-1,9′-xanthen]-3-one (***2***)

The intermediate **9** 2-(4-(but-3-yn-1-yloxy)butyl)isoindoline-1,3-dione (542.2 mg, 2 mmol) was dissolved in EtOH (10 mL). Hydrazine hydrate (981.1 mg, 20 mmol) was added dropwise and the reaction was heated to reflux for 30 min until the white solid was not increased. The solvent was removed under reduction in vacuo. Then, ethyl acetate and water were added. The water layer was adjusted to pH 3; the water phase was then collected and adjusted to pH 9 and extracted with ethyl acetate (3 × 30 mL). The combined organic phases were washed with water, dried with Na_2_SO_4_, and evaporated to get the crude primary amine 4-(but-3-yn-1-yloxy)butan-1-amine. The primary amine (253.8 mg, 1.5 mmol) was added to a suspension of Rhodamine B (956.0 mg, 2 mmol), HATU (1.141 g, 3 mmol), and Et_3_N (809.5 mg, 8 mmol) in DMF (15 mL) at room temperature for 16 h. Then, ethyl acetate and water were added. The separated aqueous phase was extracted with ethyl acetate (3 × 30 mL). The combined organic phases were washed with water, dried with Na_2_SO_4_, and evaporated to dryness. Purification was carried out with silica gel (hexane/ethyl acetate = 5:1) to get the pink solid **2**. ESI/MS: 566.33 [M + H]^+^. 1H-NMR (400 MHz, Chloroform-*d*) δ 7.88 (d, *J* = 4.6 Hz, 1H), 7.44–7.37 (m, 2H), 7.05 (d, *J* = 4.6 Hz, 1H), 6.45–6.34 (m, 4H), 6.25 (d, *J* = 8.8 Hz, 2H), 3.42–3.36 (m, 2H), 3.36–3.27 (m, 8H), 3.24–3.18 (m, 2H), 3.13 (d, *J* = 8.1 Hz, 2H), 2.38–2.29 (m, 2H), 1.91 (s, 1H), 1.34 (t, *J* = 4.3 Hz, 2H), 1.25–1.17 (m, 2H), 1.15 (s, 12H).

### 3.5. Synthesis of 7-(Diethylamino)-N-(3-ethynylphenyl)-2-oxo-2H-chromene-3-carboxamide (***3***)

3-Ethynyl-benzenamin (175.7 mg, 1.5 mmol) was added to a suspension of 7-(diethylamino)-2-oxo-2*H*-1-benzopyran-3-carboxylic acid (261.3 mg, 1 mmol), HATU (570.4 mg, 1.5 mmol), and Et_3_N (464.8 mg, 4 mmol) in DMF (7.5 mL) at room temperature for 16 h. Then, dichloromethane and water were added. The separated aqueous phase was extracted with dichloromethane (3 × 30 mL). The combined organic phases were washed with water, dried (Na_2_SO_4_), and evaporated to dryness, followed by purification by silica gel (CH_2_Cl_2_/MeOH = 100:3) to give the yellow solid **3**. ESI/MS: 361.05 [M + H]^+^, ^1^H-NMR (400 MHz, DMSO-d6) δ 10.74 (s, 1H), 8.72 (s, 1H), 7.91 (t, *J* = 1.9 Hz, 1H), 7.71 (d, *J* = 9.1 Hz, 1H), 7.60 (m, *J* = 8.3, 2.3, 1.0 Hz, 1H), 7.33 (t, *J* = 7.9 Hz, 1H), 7.18 (dt, *J* = 7.7, 1.4 Hz, 1H), 6.82 (dd, *J* = 9.0, 2.5 Hz, 1H), 6.64 (d, *J* = 2.4 Hz, 1H), 4.17 (s, 1H), 3.47 (q, *J* = 7.0 Hz, 4H), 1.12 (t, *J* = 7.0 Hz, 6H).

### 3.6. Synthesis of 7-(Diethylamino)-2-oxo-N-(prop-2-yn-1-yl)-2H-chromene-3-carboxamide (***4***)

2-Propynylamine (33.1 mg, 0.6 mmol) was added to a suspension of 7-(diethylamino)-2-oxo-2*H*-1-benzopyran-3-carboxylic acid (130.7 mg, 0.5 mmol), HATU (285.2 mg, 0.75 mmol), and Et_3_N (232.4 mg, 2 mmol) in DMF (4 mL) at room temperature for 16 h. Then, dichloromethane and water were added. The separated aqueous phase was extracted with dichloromethane (3 × 30 mL). The combined organic phases were washed with water, dried with Na_2_SO_4_, and evaporated to dryness, followed by purification by silica gel (CH_2_Cl_2_/MeOH = 50:1) to give the yellow solid **4**. ESI/MS: 299.13 [M + H]^+^. ^1^H-NMR (400 MHz, DMSO-*d*_6_) δ 8.80 (s, 1H), 8.64 (s, 1H), 7.66 (d, *J* = 9.7 Hz, 1H), 6.78 (d, *J* = 9.5 Hz, 1H), 6.58 (s, 1H), 4.05 (s, 2H), 3.28 (s, 4H), 3.10 (s, 1H), 1.10 (t, *J* = 5.4 Hz, 6H).

### 3.7. The Protocol of Base Optimization

To a mixture of BrdU (30.7 mg, 0.1 mmol), sodium d-isoascorbate (29.7 mg, 0.15 mmol), and base (0.3 mmol) in 10 mL water were added DTBPPS (5.36 mg, 0.02 mmol) and K_2_PdCl_4_ (6.53 mg, 0.15 mmol) under Ar at 37 °C. Two hours later, the probe **1** (54.1 mg, 0.1 mmol) and CuI (1.9 mg, 0.01 mmol) in 10 mL DMF were added and degassed in vacuo and purged of Ar several times. After another two hours, the reaction was ended, and water and ethyl acetate were added. The separated aqueous phase was extracted with ethyl acetate (3 × 30 mL). The combined organic phases were washed with water, dried (Na_2_SO_4_), and evaporated to dryness. Purification by silica gel (CH_2_Cl_2_/MeOH = 10:1 + 0.5% NH_4_^+^OH^−^) resulted in the pink solid **12**.

### 3.8. The Protocol of Catalytic System Optimization

Condition 1: To a mixture of BrdU (30.7 mg, 0.1 mmol), Sodium d-isoascorbate (29.7 mg, 0.15 mmol), and *n*-Bu_4_N^+^OH^−^ (77.7 mg, 0.3 mmol) in 10 mL water were added DTBPPS (5.36 mg, 0.02 mmol) and Pd(OAc)_2_ (4.5 mg, 0.02 mmol) under Ar at 37 °C. Two hours later, the probe **1** (54.1 mg, 0.1 mmol) and CuI (1.9 mg, 0.01 mmol) in 10 mL DMF were added and degassed in vacuo and purged of Ar several times. After another two hours, the reaction was ended, and water and ethyl acetate were added. The separated aqueous phase was extracted with ethyl acetate (3 × 30 mL). The combined organic phases were washed with water, dried (Na_2_SO_4_), and evaporated to dryness. Purification by silica gel (CH_2_Cl_2_/MeOH = 10:1 + 0.5% NH_4_^+^OH^−^) gave the pink solid **12**.

Condition 2: To a mixture of BrdU (30.7 mg, 0.1 mmol), sodium d-isoascorbate (29.7 mg, 0.15 mmol), and *n*-Bu_4_N^+^OH^−^ (77.7 mg, 0.3 mmol) in 10 mL water were added DTBPPS (5.36 mg, 0.02 mmol) and K_2_PdCl_4_ (6.53 mg, 0.15 mmol) under Ar at 37 °C. Two hours later, the probe **1** (54.1 mg, 0.1 mmol) and CuI (1.9 mg, 0.01 mmol) in 10 mL DMF were added and degassed in vacuo and purged of Ar several times. After another two hours, the reaction was ended, and water and ethyl acetate were added. The separated aqueous phase was extracted with ethyl acetate (3 × 30 mL). The combined organic phases were washed with water, dried (Na_2_SO_4_), and evaporated to dryness. Purification by silica gel (CH_2_Cl_2_/MeOH = 10:1 + 0.5% NH_4_^+^OH^−^) gave the pink solid **12**.

Condition 3: To a mixture of BrdU (30.7 mg, 0.1 mmol), Sodium d-isoascorbate (29.7 mg, 0.15 mmol), and *n*-Bu_4_N^+^OH^−^ (77.7 mg, 0.3 mmol) in 10 mL water were added S-phos (10.2 mg, 0.02 mmol) and K_2_PdCl_4_ (6.53 mg, 0.15 mmol) under Ar at 37 °C. Two hours later, the probe **1** (54.1 mg, 0.1 mmol) in 10 mL EtOH was added and degassed in vacuo and purged of Ar several times. After another two hours, the reaction was partly evaporated, and water and ethyl acetate were added. The separated aqueous phase was extracted with ethyl acetate (3 × 30 mL). The combined organic phases were washed with water, dried (Na_2_SO_4_), and evaporated to dryness. Purification by silica gel (CH_2_Cl_2_/MeOH = 10:1 + 0.5% NH_4_^+^OH^−^) gave the pink solid **12**.

### 3.9. Synthesis of 5-((3-(3′,6′-Bis(diethylamino)-3-oxospiro[isoindoline-1,9′-xanthen]-2-yl)phenyl)ethynyl)-1-((2R,4S,5R)-4-hydroxy-5-(hydroxymethyl)tetrahydrofuran-2-yl)pyrimidine-2,4(1H,3H)-dione (***12***)

To a mixture of BrdU (30.7 mg, 0.1 mmol), sodium d-isoascorbate (29.7 mg, 0.15 mmol), and *n*-Bu_4_N^+^OH^−^ (77.7 mg, 0.3 mmol) in 10 mL water were added S-phos (10.2 mg, 0.02 mmol) and K_2_PdCl_4_ (6.53 mg, 0.15 mmol) under Ar at 37 °C. Two hours later, the probe **1** (54.1 mg, 0.1 mmol) in 10 mL EtOH was added and degassed in vacuo and purged of Ar several times. After another two hours, the reaction was partly evaporated, and water and ethyl acetate were added. The separated aqueous phase was extracted with ethyl acetate (3 × 30 mL). The combined organic phases were washed with water, dried (Na_2_SO_4_), and evaporated to dryness. Purification by silica gel (CH_2_Cl_2_/MeOH = 10:1 + 0.5% NH_4_^+^OH^−^) resulted in the pink solid **12**. ESI/MS: 768.3593 [M + H]^+^. ^1^H-NMR (400 MHz, DMSO-*d*_6_) δ 11.64 (s, 1H), 8.29 (s, 1H), 7.86 (s, 1H), 7.54 (s, 2H), 7.14 (s, 3H), 6.61 (s, 1H), 6.53 (s, 2H), 6.35 (d, *J* = 11.5 Hz, 2H), 6.26 (s, 2H), 6.08 (s, 1H), 5.21 (s, 1H), 5.09 (s, 1H), 4.20 (s, 1H), 3.76 (s, 1H), 3.56 (d, *J* = 15.9 Hz, 2H), 1.95 (s, 1H), 1.20 (s, 1H), 1.02 (d, *J* = 10.3 Hz, 12H).

### 3.10. Synthesis of 5-(4-(4-(3′,6′-Bis(diethylamino)-3-oxospiro[isoindoline-1,9′-xanthen]-2-yl)butoxy)but-1-yn-1-yl)-1-((2R,4S,5R)-4-hydroxy-5-(hydroxymethyl)tetrahydrofuran-2-yl)pyrimidine-2,4(1H,3H)-dione (***13***)

To a mixture of BrdU (30.7 mg, 0.1 mmol), sodium d-isoascorbate (29.7 mg, 0.15 mmol), and *n*-Bu_4_N^+^OH^−^ (77.7 mg, 0.3 mmol) in 10 mL water were added S-phos (10.2 mg, 0.02 mmol) and K_2_PdCl_4_ (6.53 mg, 0.15 mmol) under Ar at 37 °C. Two hours later, the probe **2** (56.5 mg 0.1 mmol) in 10 mL EtOH was added and degassed in vacuo and purged of Ar several times. After another two hours, the reaction was partly evaporated, and water and ethyl acetate were added. The separated aqueous phase was extracted with ethyl acetate (3 × 30 mL). The combined organic phases were washed with water, dried with Na_2_SO_4_, and evaporated to dryness. Purification by silica gel (CH_2_Cl_2_/MeOH = 10:1 + 0.5% NH_4_^+^OH^−^) gave the pink solid **13**: ESI/MS: 792.3911 [M + H]^+^. ^1^H-NMR (400 MHz, DMSO-*d*_6_) δ 11.52 (s, 1H), 8.06 (s, 1H), 7.77–7.69 (m, 1H), 7.46 (p, *J* = 5.4 Hz, 2H), 6.99 (d, *J* = 5.3 Hz, 1H), 6.32 (d, *J* = 10.3 Hz, 4H), 6.25 (d, *J* = 8.5 Hz, 2H), 6.10–6.04 (m, 1H), 5.20 (s, 1H), 5.02 (s, 1H), 3.5-4.5(m,8H) 3.31 (m, 8H), 3.12–3.05 (m, 2H), 2.98–2.89 (m, 2H), 2.07 (s, 1H), 2.05 (s, 1H), 1.19 (s, 2H), 1.13 (s, 2H), 1.03 (d, *J* = 6.6 Hz, 12H).

### 3.11. 7-(Diethylamino)-N-(3-((1-((2R,4S,5R)-4-hydroxy-5-(hydroxymethyl)tetrahydrofuran-2-yl)-2,4-dioxo-1,2,3,4-tetrahydropyrimidin-5-yl)ethynyl)phenyl)-2-oxo-2H-chromene-3-carboxamide (***14***)

To a mixture of BrdU (30.7 mg, 0.1 mmol), sodium d-isoascorbate (29.7 mg, 0.15 mmol), and *n*-Bu_4_N^+^OH^−^ (77.7 mg, 0.3 mmol) in 10 mL water were added S-phos (10.2 mg, 0.02 mmol) and K_2_PdCl_4_ (6.53 mg, 0.15 mmol) under Ar at 37 °C. Two hours later, the probe **3** (36.0 mg, 0.1 mmol) in 10 mL EtOH was added and degassed in vacuo and purged of Ar several times. After another two hours, the reaction was partly evaporated, and water and dichloromethane were added. The separated aqueous phase was extracted with dichloromethane (3 × 30 mL). The combined organic phases were washed with water, dried with Na_2_SO_4_, and evaporated to dryness. Purification by silica gel (CH_2_Cl_2_/MeOH = 10:1 + 0.5% NH_4_^+^OH^−^) gave the yellow solid **14**: ESI/MS: 587.0389 [M + H]^+ 1^H-NMR (400 MHz, DMSO-*d*_6_) δ 10.76 (s, 1H), 8.72 (s, 1H), 8.36 (s, 1H), 7.91 (s, 1H), 7.71 (d, *J* = 9.1 Hz, 1H), 7.57 (d, *J* = 8.2 Hz, 1H), 7.35 (t, *J* = 7.9 Hz, 1H), 7.02 (d, *J* = 8.6 Hz, 1H), 6.83 (s, 1H), 6.64 (s, 1H), 6.10 (t, *J* = 6.5 Hz, 1H), 5.23 (s, 1H), 5.14 (s, 1H), 4.23 (s, 1H), 3.77 (s, 1H), 3.47 (s, 4H), 3.34 (m, *J* = 7.0 Hz, 2H), 2.14 (s, 1H), 1.71 (s, 1H), 1.31 (s, 6H).

### 3.12. 7-(Diethylamino)-N-(3-(1-((2R,4S,5R)-4-hydroxy-5-(hydroxymethyl)tetrahydrofuran-2-yl)-2,4-dioxo-1,2,3,4-tetrahydropyrimidin-5-yl)prop-2-yn-1-yl)-2-oxo-2H-chromene-3-carboxamide (***15***)

To a mixture of BrdU (30.7 mg, 0.1 mmol), sodium d-isoascorbate (29.7 mg, 0.15 mmol), and *n*-Bu_4_N^+^OH^−^ (77.7 mg, 0.3 mmol) in 10 mL water were added S-phos (10.2 mg, 0.02 mmol) and K_2_PdCl_4_ (6.53 mg, 0.15 mmol) under Ar at 37 °C. Two hours later, the probe **4** (29.8 mg, 0.1 mmol) in 10 mL EtOH was added and degassed in vacuo and purged of Ar several times. After another two hours, the reaction was partly evaporated, and water and dichloromethane were added. The separated aqueous phase was extracted with dichloromethane (3 × 30 mL). The combined organic phases were washed with water, dried with Na_2_SO_4_, and evaporated to dryness. Purification by silica gel (CH_2_Cl_2_/MeOH = 10:1 + 0.5% NH_4_^+^OH^−^) resulted in the yellow solid **15**: ESI/MS: 525.2305 [M + H]^+^. ^1^H-NMR (400 MHz, DMSO-*d*_6_) δ 11.58 (s, 1H), 8.85 (s, 1H), 8.65 (s, 1H), 8.14 (s, 1H), 7.66 (d, *J* = 9.3 Hz, 1H), 6.78 (d, *J* = 11.5 Hz, 1H), 6.59 (s, 1H), 6.06 (s, 1H), 5.19 (s, 1H), 5.05 (s, 1H), 4.29 (s, 2H), 4.18 (s, 1H), 3.74 (s, 1H), 3.55 (s, 1H), 3.52 (s, 1H), 3.45 (d, *J* = 7.1 Hz, 4H), 2.08 (s, 1H), 2.06 (s, 1H), 1.09 (d, *J* = 6.7 Hz, 6H).

## 4. Conclusions

As an important C5-modified uridine, BrdU has been widely used to investigate DNA replication in biological studies. It is desirable to develop a chemical method to label BrdU. In this study, we applied the well-known Sonogashira reaction to label BrdU with fluorescent probes. We screened various conditions including the base and the catalyst system in the aqueous medium, and describe mild Sonogashira reaction conditions (K_2_PdCl_4_, S-Phos, *n*-Bu_4_N^+^OH^−^, Sodium d-isoascorbate, EtOH/H_2_O = 1:1, 37 °C, Ar) for BrdU fluorescent labeling. In addition, we also discovered that there is no obvious difference in the labeling rate when the fluorescent probe contains either aliphatic or aryl alkynes.

## Figures and Tables

**Table 1:** Optimization of the base used in the Sonogashira reaction under typical reaction conditions.

Base	Yield (%)
Na_2_CO_3_	19
Cs_2_CO_3_	21
Et_3_N	28
Quinine	34
DABCO	39
*n*-Bu_4_N^+^OH^−^	42

**Table 2:** The effect of catalyst systems in the Sonogashira reaction.

The Catalyst	The Solvent	Yield
Pd(OAc)_2_, DTBPPS, CuI	DMF/H_2_O = 1:1	22%
K_2_PdCl_4_, DTBPPS, CuI	DMF/H_2_O = 1:1	42%
K_2_PdCl_4_, S-phos	EtOH/H_2_O = 1:1	75%
